# Supplementary material for: Estimating the Carbon Footprint of Healthcare in the Canton of Geneva and Reduction Scenarios for 2030 and 2040
Source: Int J Environ Res Public Health. 2024 May 28;21(6):690. doi: 10.3390/ijerph21060690 (PMC11204316; doi:10.3390/ijerph21060690)

International Journal of *Environmental Research and Public Health*

Article

Estimating the carbon footprint of healthcare in the canton of Geneva and reduction scenarios for 2030 and 2040

Supplementary Materials:

Figure S1: Greenhouse gases emissions breakdown of the Hospitals sub-sector

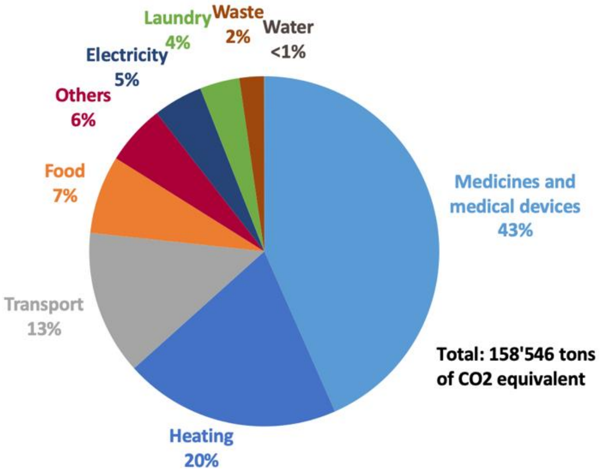

Figure S2: Greenhouse gases emissions breakdown of the Pharmacies sub-sector, without medicines

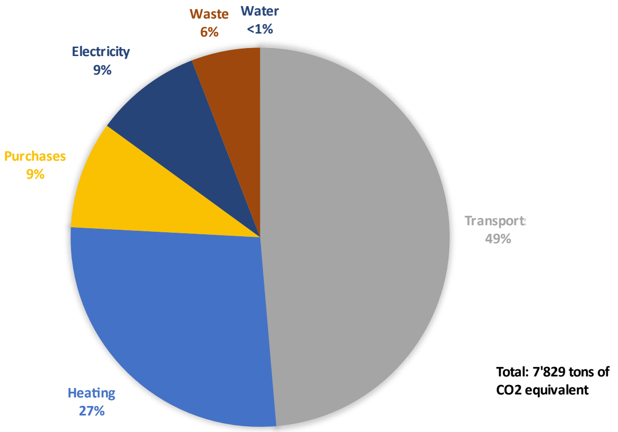

Figure S3: Greenhouse gases emissions breakdown of the Pharmacies sub-sector, with medicines

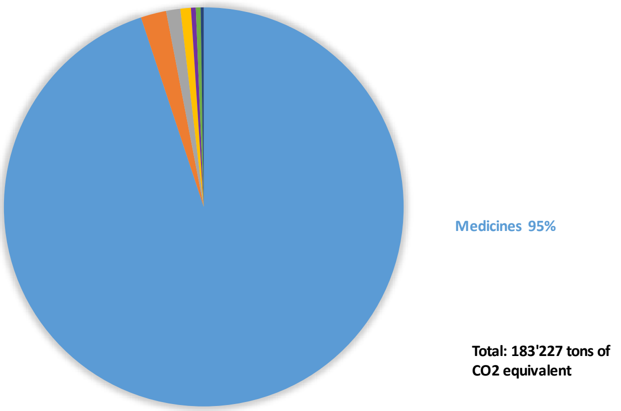

Figure S4: Greenhouse gases emissions breakdown of the nursing homes sub-sector

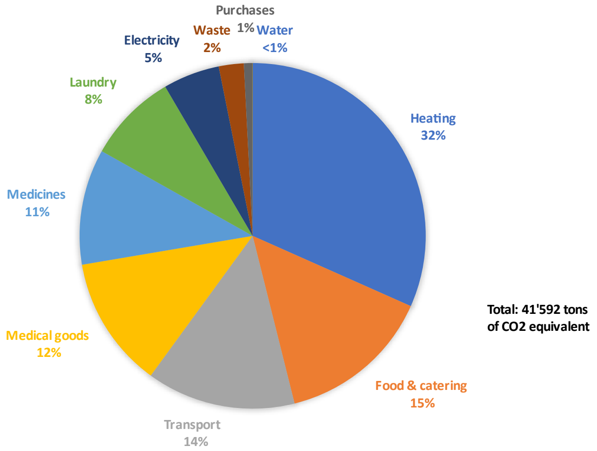

Figure S5: Greenhouse gases emissions breakdown of the medical offices sub-sector

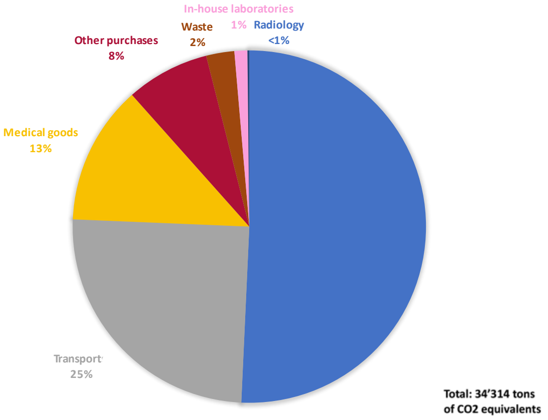

Figure S6: Greenhouse gases emissions breakdown of the homecare institution sub-sector

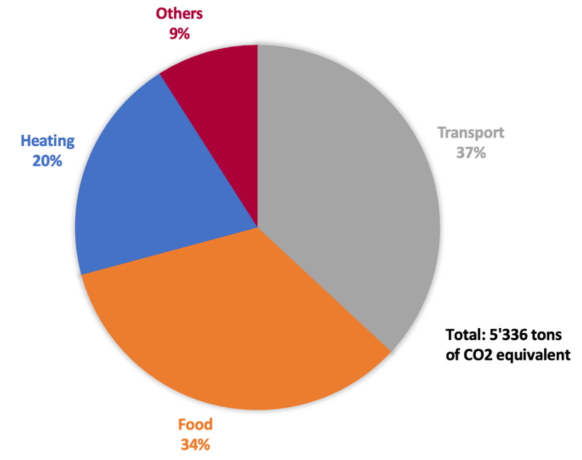

Figure S7: Greenhouse gases emissions breakdown of the ambulance sub-sector

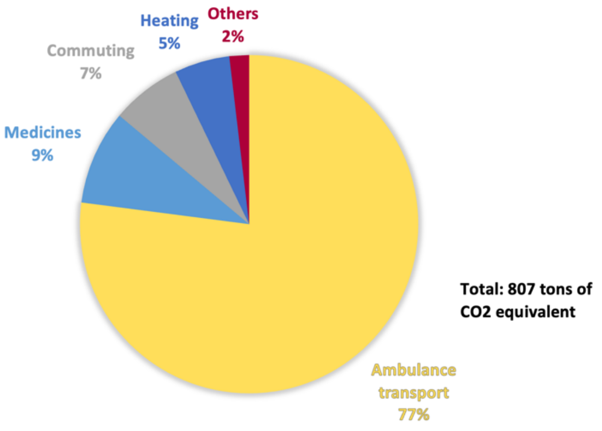

Supplement: Supplementary file 1 [file ijerph-21-00690-s001.zip › ijerph-2973701-supplementary.pdf]
